# Pick me, pick me: Proposed indicators for collaboration in medical education research

**DOI:** 10.1111/medu.15691

**Published:** 2025-05-20

**Authors:** Joanna Tai, Rola Ajjawi, Gabrielle Maria Finn

**Affiliations:** ^1^ Centre for Research in Assessment and Digital Learning (CRADLE) Deakin University Melbourne Victoria Australia; ^2^ The University of British Columbia Vancouver British Columbia Canada; ^3^ School of Medical Sciences, Faculty of Biology, Medicine, and Health University of Manchester Manchester UK

## Abstract

Collaboration is a regular part of medical education research. There has been minimal formalised unpicking of features which may promote enjoyable and effective collaboration. This paper focuses on the social qualities of individuals to highlight important aspects to consider when assembling collaborative research teams. We draw on the concept of persona from celebrity studies research to formulate five Famousness Dimensions (FD). Then we use a broader range of education and sociological theories to identify contextual aspects of collaboration, collectively termed KRUD: knowing‐that/knowing‐how, relevant experience, (Un)kindness and demands of day jobs. Together, these form the Proposed Indicators for Collaboration in Medical Education research (PIC‐ME). Although developed with tongue‐in‐cheek intent, the PIC‐ME may facilitate holistic judgements of collaborative potential, and offer a mechanism for shared understanding towards international collaboration that sparks joy and furthers medical education research.

## Abstract 2

The Proposed Indicators for Collaboration in Medical Education research (PIC‐ME) offers new considerations for assembling your team that might just help smooth tricky research collaborations. #MedEducUnleashed

## 1 INTRODUCTION

Collaboration has long been examined within medical education research as a means of both improving interdisciplinary teamwork within health care^1^ and research processes themselves.^2^ In research collaboration, working effectively in teams is important to make the most of scarce funding, and to support researcher development. When collaboration does not go well, we become frustrated: much time and effort can be wasted. The post‐covid working environment has fostered hybrid connections, with greater opportunities and expectations for research collaboration. Given this intensification, and since we are all human with finite physical and mental resources, it is important to consider which collaborations we should participate in, not just for strategic purposes, but also for our joy and wellbeing.

An assessment of collaborative potential is required. Previous work has predominantly focussed on individuals within the collaboration; this research suggests collaboration could be conceptualised as a psychometric property, tasks or activities or ‘togetherness’,^1^ and a typology of collaboration roles has been developed.^2^ In this paper, we argue that social contexts and collective properties of the team are equally important for success. Therefore, we have developed a set of Proposed Indicators for Collaboration in Medical Education research (PIC‐ME), encompassing individual famousness and contextual considerations. In the following sections, we elaborate on each aspect in turn.

## 2 INDIVIDUAL FAMOUSNESS: A CELEBRITY STUDIES PERSPECTIVE

When identifying *who* to collaborate with in research and optimal group configuration, the social presence of and interactions between individuals should be considered. Within medicine, brand identity^3^ and Goffman's dramaturgical model of self‐presentation^4^ have recently been used to unpack physician identity, with research collaboration a subsidiary consideration. The Kardashian Index, while more research focussed, was formulated to identify discrepancies between social media presence and publication record,^5^ but this focuses on output metrics, rather than the qualitative experience of collaboration. We take a different approach, drawing from Celebrity Studies, to apply the concept of persona to researchers. Persona studies evolved from studying celebrities' strategic identity construction to a wider exploration of how ‘regular humans’ present themselves and navigate the public world.^6^ We apply this understanding to research personas in medical education. Personas are a strategic identity, defined as: ‘a fabricated reconstruction of the individual that is used to play a role that both helps the individual navigate their presence and interactions with others and helps the collective to position the role of the individual in the social’ (p. 1).^7^ Persona‐making is a practice with five dimensions,^8^, ^9^ each of which we introduce and then translate for research collaboration.

### 2.1 Public

The *public dimension* refers to the ‘extended social network of the individual that includes personal friends, professional associates, plus their networks, and the systems and platforms that connect them’ (p. 2).^9^ This spectrum of publicness might range from a tight group of friends to a massive global audience. Publicness concerns how individuals curate and network and meet people at conferences, through work, symposia and presentations and, of course, social media. This is the “do you know who I am?” aspect. As researchers become more famous through talking about their work, their publicness expands. They become more known by others, which has a snowball effect of attracting more research collaborators (including the most prized for promotion to professor – the international variety of collaborator!). This dimension may explain why ‘networking’ is viewed as an important skill to develop, and the importance of conference attendance and speaking engagements.

### 2.2 Mediatised

The second dimension relates to the mediatised quality of the online persona, which accumulates piece by piece over time, through digital (and physical) records, which are likely out of an individuals' control. Although this was ‘once only the province of individuals in film, television, print, and radio’ (p. 8),^9^ researchers need to consider how we “market” ourselves, and support more junior researchers to “get their name out there”. The longer the career, the more records exist, and these may have an enduring impact on others' perceptions (both good and bad). One's persona can be communicated in many media formats beyond written ‘voice’^10^ in traditional boring long papers (which, in this attention economy,^11^ are a poor form of currency) – consider how newsletters, videos, podcasts, blogs, selfies or even becoming a viral sensation contribute to being known. These accessible and tangible records of one's expertise may lead to more approaches from others.

### 2.3 Performativity

The public performance of the self is not entirely real, nor is it wholly fictional. Instead, it is a collection of intentionally staged performances which may also become ritualised.^9^ This performance spans previous, current and future work, from overt messaging like interviews or talks to more subtle cues, like voluntary service such as reviewing papers (which gets you known to editors). Researchers may also have alter egos to present, such as student‐facing (the kind professor) or institution‐facing (the good academic citizen). Although individuals can curate their presented ‘self’ by placing a spin on their narrative for particular people on different platforms, the audience also interprets this performance. Thus, it is important to take performative metrics with a grain of salt: an h‐index, citations and even impact measures are to a degree indicative of time in the field, rather than wholly representing famousness. Any insights into the ‘backstage’ researcher may be revelatory as to who you actually want on your team. Ideally, espoused values and actions ‘backstage’ and ‘on show’ should align, and we should be the change we want to see.

### 2.4 Collective

The fourth dimension of persona is one that ‘works to produce, seek out, and move between connections, resulting in a collective’ (p. 5).^9^ The use of the term ‘collective’ in considering online cultures avoids normative notions of community, instead suggesting patterns of relations within particular groups. This notion of collective identity prompts consideration of one's research networks. Who are your ‘peeps’? Which professional associations or networks are you a part of, and which platforms (e.g. LinkedIn, Bluesky) do you use to stay in touch with others? How many degrees of separation exist between potential collaborators? Reflecting on connections can help understand one's own perceived positioning, as well as identifying future collaborators who are likely to share interests.

### 2.5 Value

This dimension considers the intention and strategy behind creating a persona and is made up of the elements of agency, reputation and prestige. These are the *whys* that motivate individuals to proceed in particular directions, cultivating certain aspects and perceptions more than others to attract attention or build respect. This could be translated as what Popkewitz^12^ terms commitments and dispositions that drive a researcher's paradigmatic view, and offer the potential for shared vision among collaborators.

Cultivating a research persona using the five dimensions may increase one's own collaborator value. Indeed, we might consider prominent medical education researchers and the personas they have intentionally or unconsciously constructed: from warm and welcoming, to distant and intimidating, and the impact this has on invitations to collaborate.

Together, these dimensions can inform decisions about inviting collaborators: alignment of values and collectives is important, and a critical examination is made of a researcher's performances, media outputs and public information. But let us not be completely cynical, that famousness is the only desirable quality for a researcher. Although fame can increase prestige on a grant application or satisfy diversity requirements, for good research, genuine engagement is important. Though there may be a correlation between fame and success, a causal relationship cannot be relied upon.

## 3 CONTEXTUAL CONSIDERATIONS: THE KRUD

Context matters. And, as with everything else, context influences the effectiveness of our research personas. Collectively, we call these contextual considerations the “KRUD”, an acronym devised of the following: Knowing‐that/knowing‐how, Relevant experience, (Un)kindness and Demands of day jobs. We introduce these based on our collective experience as collaborators, and drawing on the educational, psychological and sociological literatures.

### 3.1 Knowing‐that and knowing‐how

Innovative ideas and perspectives are crucial to inspire the team. Simultaneously, there also needs to be know‐how^13^, ^14^: that is, collaborators who can roll up their sleeves and get things done, with institutional street‐smarts to navigate in and around systems effectively. Across a team there should be a sufficiency of both deep thinking *and* logistical expertise. This may be [un]evenly distributed across team members, in which case, cross‐consideration with ‘demands of day jobs’ should be undertaken.

### 3.2 Relevant and related experience

This includes subject matter expertise, methodological expertise, lived experience expertise, practical stakeholder and/or significant intellectual interest. Diversity of background and experience should be considered across the team. Opportunities to further develop expertise through the doing of the project is also important; this is key to ensure ongoing motivation to participate. Understanding which of these are intrinsic motivators for collaborators could help to ensure they remain committed to the project.^15^, ^16^

### 3.3 (Un)kindness

The ability to take risks within a team is important for learning and functionality.^17^ This requires a supportive environment where different approaches can be floated, without fear of being shut down. It is also important to be able to own mistakes and seek help (or simply admit that we have not done our allocated tasks). The team must consider how to move from being an intimidating bunch of individuals to a pleasant group environment. For new collaborations, collateral information about past performance from those external to the team may be helpful: does anyone have exacting standards and a short temper, or a particular worldview? Who will be the snack‐provider and social glue within the team?

### 3.4 Demands of day jobs

Collaborators will have a range of commitments and responsibilities which influence how they contribute and interact within the collaboration. The collaboration may also offer respite from organisational tribulations. This two‐way interplay between project‐specific and general demands can significantly impact contributions. Support from bosses, alignment with national or institutional strategies and goals, and future job and career aspirations of collaborators may also need to be taken into account in understanding why someone has not read the email or met that deadline … again.

## 4 HOLISTIC JUDGEMENT FOR PIC‐ME

The famousness dimensions (FD) and the KRUD combine to form the Proposed Indicators for Collaboration in Medical Education, i.e. “PIC‐ME”. We encourage collaborative use of the PIC‐ME as part of team formation and evaluation. Relevant information for each collaborator should be noted against the FD and KRUD. Then, overall, the PIC‐ME requires a holistic judgement to be made. One of three ordinal ratings should be applied: “could be a disaster but worth it”, “we will need to put in effort” and “fab and glam, darling” (thanks to Dr Jonas Nordquist of the Karolinksa Institutet for this phrase). Group members can initially reflect on and make a judgement in relation to the team, and then collectively determine if collaboration is a good idea. To be clear, PIC‐ME does not form a psychometric construct: descriptive information should not be summed together but instead considered holistically. Although more famousness might be perceived as better, there can also be drawbacks where collectives and values across the team are not in harmony: there may be tensions regarding leadership and decision‐making, leading to ruptures and abandoned projects. Some consideration should also be given to how a collaboration might best proceed across international borders, and high and low‐resource settings to promote inclusivity in health professions education.^18^ A group that rates itself disastrous should not proceed with the collaboration.

## 5 DISCUSSION & CONCLUSIONS

In this work, we have playfully proposed that the choice of collaborator should be determined from more than just the sum of individual metrics like the h‐index or the Kardashian index. Our underlying contentions are however in earnest: beyond the individual, collective, embodied and enacted actions within the social world (which make up day‐to‐day research endeavours) are important. Holistic judgements should be applied to develop a shared understanding of collaborative power, which could perhaps be called a ‘hall of fame’. In articulating matters of fame in research, we seek to offer an explanatory vocabulary for some social aspects of collaboration. Junior and early career researchers should, therefore, not be dissuaded from engaging with more famous members of the medical education communities. Rather, we hope this paper offers tools to understand ways of connecting with collaborators to achieve successful research collaborations. There are of course limitations to this work. Although we have collaboratively developed these criteria, there may be other important aspects which are not articulated in this paper. We welcome further discussion of the indicators and would even like to collaborate with others to refine this offering.

## AUTHOR CONTRIBUTIONS

**Joanna Tai:** Conceptualization; writing—original draft; writing—review and editing; project administration. **Rola Ajjawi:** Conceptualization; writing—review and editing; writing—original draft; supervision. **Gabrielle Maria Finn:** Conceptualization; writing—review and editing; supervision.

## CONFLICT OF INTEREST STATEMENT

The authors have no conflicts of interest to declare.

Submission for the Medical Education Unleashed issue.

## ETHICS STATEMENT

This paper does not contain empirical research data and, therefore, no ethics approval process was undertaken.

## Data Availability

Data sharing not applicable to this article as no datasets were generated or analysed during the current study.
